# The effect of *Zingiber Officinale* Extract on Preventing Demyelination of Corpus Callosum in a Rat Model of Multiple Sclerosis

**DOI:** 10.52547/ibj.2979

**Published:** 2022-08-27

**Authors:** Valiollah Moradi, Ebrahim Esfandiary, Mustafa Ghanadian, Nazem Ghasemi, Bahman Rashidi

**Affiliations:** 1 Department of Anatomical Sciences, School of Medicine, Isfahan University of Medical Sciences, Isfahan, Iran;; 2 Department of Pharmacognosy, Faculty of Pharmacy and Pharmaceutical Sciences, Isfahan University of Medical Sciences, Isfahan, Iran

**Keywords:** Corpus callosum, Demyelination, Ginger extract, Multiple sclerosis, Rat

## Abstract

**Background:**

Multiple sclerosis is the most prevalent neurological disability of young adults. Anti-inflammatory drugs have relative effects on MS. The anti-inflammatory and antioxidative effects of *Zingiber officinale *(ginger) have been proven in some experimental and clinical investigations. The aim of this study was to evaluate the effects of ginger extract on preventing myelin degradation in a rat model of MS.

**Methods:**

Forty nine male Wistar rats were used in this study and divided into four control groups: the normal group, cuprizone-induced group, sham group (CPZ + NaCMC), standard control group (fingolimod + cuprizone), including three experimental groups of CPZ, each receiving three different doses of ginger extract: 150, 300, and 600mg/kg /kg/day.

**Results:**

Ginger extract of 600 mg/kg prevented CC from demyelination; however, a significant difference was observed in the fingolimod group (p < 0.05). Difference in the CPZ group was quite significant (p < 0.05).

**Conclusion:**

Treatment with ginger inhibited demyelination and alleviated remyelination of corpus callosum in rats. Therefore, it could serve as a therapeutic agent in the MS.

## INTRODUCTION

Multiple sclerosis is a chronic neurological disorder of the CNS that causes damage to the axonal myelin sheath^[1,2]^. After myelin degradation, numerous plaques are formed around nerve fibers and the lack of myelin leads to disturbances in the direction of neural impulses^[3,4]^. On average, five persons out of every 100,000 people are diagnosed with this disease worldwide^[5]^. The neuropathic disorder is more common among young people, with a high prevalence in women than men^[6,7]^. 

More than 2.5 million people are affected by MS all over the world, with an increasing trend at an alarming rate^[8]^. In many countries, especially Western societies, MS is the main risk factor of disability^[9]^. It has been discovered that both environmental and genetic factors play a role in the prevalence and geographical dispersion of this disease^[10,11]^. The exact mechanism of neuronal loss remains a challenge for the scientific community and is still unclear^[12]^. 

MS is a chronic inflammatory, demyelinating and neurodegenerative disorder^[13,14]^. Inflammation in the CNS is induced by the entry of lymphocytes. T cells secrete proinflammatory molecules that increase the permeability of the BBB and the entrance of other immune cells^[15]^. There is no effective clinical treatment or therapy for MS disease till date^[16,17]^. The available medications have been shown to be associated with many side effects, including headaches, back pain, macular edema, high risk of serious infections, diarrhea, hair thinning, and so forth^[18]^. Medicinal plants have long been used for therapeutic purposes^[19,20]^, and according to the World Health Organization approximates, 80% of the world’s population tends to use herbal medicines^[21]^.

*Zingier officinale* Roscoe (Ginger) is a tropical and subtropical plant that is widely used as a spice^[22]^ and is native to Southeastern Asia. Ginger has been used for thousands of years for the treatment of numerous diseases^[23]^ or as an antiemetic^[24]^. It has analgesic^[25]^, antitumor^[26]^, antioxidant, and anti-inflammatory impacts^[27]^. Ginger has several effective ingredients, including gingerols, shogaols, and some phenolic ketone derivatives^[28]^. Among the ginger compounds, 6-gingerol has been demonstrated to modulate neuroinflammation and prevent cognitive impairment in animal models^[29]^. In addition, this compound has significant potential as a novel anti‐inflammatory agent for treating autoimmune diseases via direct modulatory effects on dendritic cells^[30]^. Treatment of EAE mice with ginger extract downregulates the expression of inflammatory chemokines^[31]^. As a result, in patients with Alzheimer’s and other neurological diseases, 6-Shogaol might improve the symptoms of the diseases^[32,33]^. In addition, neuroprotective effects of 6-Shogaol and its metabolite in the remyelination of the spinal cord have been reported in an MS model, as well^[2]^.

Considering the use of nutritional therapy and medicinal properties of ginger, especially its anti-inflammatory properties, this study aimed to evaluate the effect of ginger extract on preventing myelin degradation in a rat model of the MS. Additionally, the effect of ginger extract on MS was compared with fingolimod, which is used for the treatment of MS.

## MATERIALS AND METHODS

**Animals**

All experiments on rat models were conducted at the Central Laboratory of Isfahan University of Medical Sciences. Male Wistar rats (n = 94) of eight weeks old, weighing 150-200 g, were purchased from the Pasteur Institute, Tehran, Iran and maintained in the light/dark (12: 12) cycle at a constant temperature in polypropylene boxes with free access to food and tap water. The rats were divided into seven groups (seven rats in each group). The first four groups were considered as control, including (1) the normal group that was not subjected to any intervention (Cont.), (2) the M.S group including CPZ-induced MS rats that did not receive any treatment (Cup); (3) sham group consisting of rats with MS that received NaCMC as the fingolimod and ginger solvent; (4) standard treatment group: M.S + fingolimod treatment (0.5 mg/kg/day) and three experimental groups as follows: (1) M.S + ginger extract (150 mg/kg/day; low extract (ExL), (2) M.S + ginger extract (300 mg/kg/day; medium extract (ExM), and (3) M.S + ginger extract (600 mg/kg/day; high extract (ExH). Treatment with fingolimod and ginger extract was initiated one week before the induction of MS and lasted for four weeks after MS induction. All the extracts and other drugs were administered by oral gavage.

**Preparation of ethanolic extract of ginger**

The rhizomes of ginger were purchased from an herbal medicine market in Isfahan (Iran). The ginger was identified, authenticated, and its extract was prepared under the supervision of Dr. Ghanadian, an academic staff of the Department of Pharmaceutical Chemistry and Pharmacognosy, Isfahan University of Medical Sciences, Isfahan, Iran. Initially, 20 kg of fresh ginger rhizome was air-dried in shade and turned into powder mechanically (2.45 kg), and then its extract was prepared by maceration using 70% ethanol as a solvent for four days, repeated three times. The collected extracts were combined and concentrated using a rotary evaporator (Heidolph Instruments Gmbh & Co., Germany) at 45 °C and low pressure (50 mbar) to obtain a gummy ethanol-free extract. The gummy extract was lyophilized by a freeze-dryer (Snijders, Tilburg, Netherlands) at -50 °C and 0.250 mbar for 48 h to obtain the dried powder (355 g). The extract yield was 14.5% of the dried weight of plant material.

**Extract standardization by the determination of total polyphenols**

Ginger extract was standardized by the determination of total phenolic content through the colorimetric method and Folin-Ciocalteau according to the mg of GAEs. A standard calibration curve was prepared against 10 different concentrations of gallic acid as the standard. Subsequently, 20 μL of each concentration of the standard solution was taken, mixed with 1.58 mL of water and 100 μL of Folin–Ciocalteau reagent and shaken for 10 min. Also, 300 μL of the saturated NaHCO_3_ solution was added to the mixture, and after 2 h, the OD absorbance was measured at 765 nm by an ultraviolet/visible spectrophotometer. Following the preparation of the gallic acid standard curve, the total extract and blank were analyzed as mentioned above in triplicates^[33]^.

**CPZ** **-** **induced demyelination**

An MS model was induced in all experimental groups, except for the control group. For this purpose, 0.6% CPZ (bis-cyclohexanone oxaldihydrazone, Sigma-Aldrich Inc. (USA) dissolved in corn oil was administered daily by oral gavage for four weeks.

**Basket test**

Before sampling and sacrificing the animals, the basket test was performed twice a day to determine the motor function of the animals. This test is useful in assessing motor coordination and sensorimotor deficits in rodent models of CNS disorders. A rat was placed in the center of a mesh-like basket (height: 50 cm and width: 25 cm), and then the basket was inverted quickly and carefully. The rats’ performance was evaluated by recording the latency to fall within 180 s^[34]^.

**Cardiac perfusion and sampling**

Four weeks after the induction of MS, animals were anesthetized using a combination of ketamine (100 mg/kg) and xylene (10 mg/kg). Through a midline incision, the chest was opened, and the heart was separated from the pericardium. The descending aorta was clamped, and the left ventricle was cannulated to inject the normal saline and fixative. The right atrium was opened to drain the blood. Accordingly, blood was removed, then the normal saline flow was interrupted and a fixative injection solution containing 1.6% glutaraldehyde in phosphate buffer (0.12 M; pH 7.4) was established. The skull was opened, the brain was carefully removed, and CC was isolated from the brain.

**TEM examination**

A piece of CC was isolated and postfixed in the aforementioned fixative solution at +4 °C overnight. After the preparation of the blocks, samples were sectioned using an ultramicrotome (Leica Microsystem Company, Germany) to about 90 nm thick. Sections were rinsed with phosphate buffer saline (0.12 M; pH 7.4) and postfixed with osmium tetroxide solution (1%) in PBS for 60 min and dehydrated. Uranyl acetate (2%) and Reynold’s lead citrate (Reynolds, ES 1963) were utilized to contrast the sections. Photographs of the CC axons cut in cross-section were taken, and quantification of the myelinated axons was performed on 10 images (×3000) per specimen. They were then analyzed with Digimizer image analysis software v. 5.3.5 (copyright© 2005-2019 MedCalc software). The myelin thickness was measured using at least 50 axons per sample.

**Real-time PCR analysis**

This method was used to evaluate the expression level of Olig2 and M*bp* genes in CC. Olig2 cells, as an oligodendrocyte precursor cell marker, have previously been studied^[35]^. M*bp* plays a role in the formation and compaction of the myelin sheath. Samples were isolated and snap-frozen in liquid nitrogen and stored at -80 °C. Total RNA was extracted using the BioFACT™ Total RNA Prep Kit (Biofact, Korea) according to the instructions provided by the manufacturer. To remove DNA from the samples, extracted RNA was treated with the RNase-free DNase kit (Qiagen, Germany). RNA quantity and quality were determined using spectrophotometry at 260 nm and 280 nm. Primers used in this study were designed by AlleleID 7.6 and checked against the rat genome using the BLAST website (http://blast.ncbi.nlm.nih.gov/ Blast.cgi). GAPDH was considered a housekeeping gene to normalize the gene expression. All primer sequences for GAPDH, Olig2, and M*bp* are listed in Table 1. In the next step, complementary DNA was synthesized using the BioFact™ RT-Kit (Biofact). Real-time PCR was carried out using BIOFACT™ 2× Real-Time PCR Master Mix (High ROX) along with specific primers as mentioned above and performed on a StepOnePlus system (Applied Biosystems, South Korea). The relative expression level of the target genes was calculated using the 2^-^^ ΔΔct ^method. 

**ELISA for Olig2 and MBP proteins**

Rats were sacrificed, and their brains were removed and stored at -70 °C. At the time of the ELISA examination, CC was isolated from the brain, then the tissue samples were homogenized by a homogenizer. The expression levels of Olig2 and MBP proteins were measured by the standard ELISA methods. A direct competitive ELISA technique was used for MBP. The antibody was coated in the bottom of the wells. Horse reddish peroxidase competes with MBP (main antigen) in binding to the antibodies present on the bottom of

the wells. The higher the amount of MBP antigen, the more antibody will be covered by the it. Therefore fewer antibodies will be covered by horse reddish peroxidase, resulting in decrease in OD. On the other hand, an indirect ELISA technique was used for Olig2. A 50-µL sample containing Olig2 antigen was added and coated in each well. After an overnight incubation, the wells were washed two times and added with 200 µL of blocking agent (bovine serum albumin), followed by overnight incubation of the wells in a refrigerator. The next morning, the wells were washed four times, and then a 100-µL primary Olig2 antibody was added. The ELISA plates were washed again, and 100 µL of tetramethylbenzidine substrate solution was added to the wells and incubated at room temperature for 15 min. Finally, OD values was measured in each group and then compared between them. ELISA reader was measured by using a spectrophotometric microplate reader (Bio-Rad 680,USA).

**Table 1:** Sequence of the primers used in this study

**Gene**	**Forward** ** primer**	**Reverse** ** primer**	**PCR size (bp)**
*Gapdh*	ATGACTCTACCCACGGCAAG	GGAAGATGGTGATGGGTTTC	87
*Olig2*	CACAGGAGGAACCGTGTCCT	GGTGCTGGAGGAAGATGACT	145
*Mbp*	TCACAGAAGAGACCCTCACAGC	GAGTCAAGGATGCCCGTGTC	116

**Statistical analysis**

Statistical analysis was carried out using the software SPSS (IBM, SPSS Statistics Version 24). The results of the experiments were expressed as mean ± standard error of the mean (SEM). The analysis was performed using the one-way analysis of variance (ANOVA), followed by the LSD post-hoc test. Statistical significance was defined at *p* < 0.05.

## RESULTS

Total phenolic measurement standardized extract y 

Galic acid standard curve was plotted in the concentration range of 0.5-5 μg/mL. The regression equation was expressed as *y = 0.0649x + 0.0168,* where *y*axis indicates OD absorption, and *x* axis represents GAE phenol content in the sample (μg/mL) with the correlation factor of r² = 0.9921. The lyophilized extract powder was standardized to have 8.18 ± 1.61% mg GAE /100 mg (Fig. 1).

**Rat behavioral test results improved after ginger treatment**

**Fig. 2:**
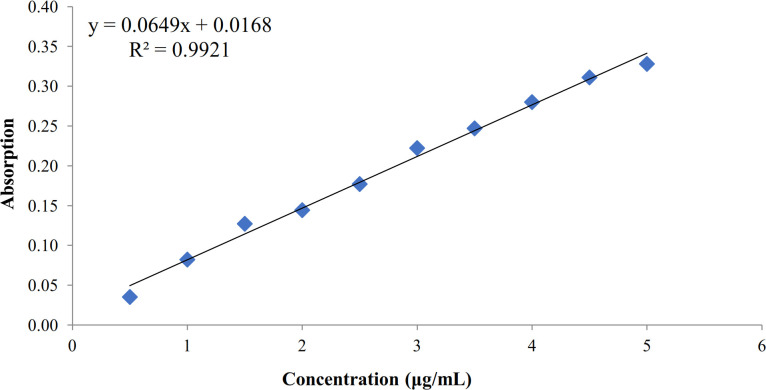
Comparison of the basket test results of the effect of ginger extract groups with different studied groups. The figure shows that the latency to fall in all the CPZ groups significantly reduced compared to the control groups (*p* < 0.001). In groups treated with fingolimod and a high dose of *Zingiber officinale* extract, the latency significantly increased (*p* < 0.001). Control group (Cont.), CUP group, Sham group, fingolimod group (Fing); ExL, ExM, and ExH, *Zingiber officinale* extract groups (150, 300, and 600 mg/kg/day, respectively; ^*^Significant difference with the control group; ^#^significant difference with the CPZ and sham groups.

The results obtained from the basket test showed that the falling down time of the basket wall increased more in ginger-treated groups that in the CPZ groups (*p* ˂ 0.01; Fig. 2). The high dose of ginger extract improved the falling down time but showed no significant difference in relation to the first group. The high dose of extract was more effective than fingolimod; however, there was no significant difference between them. According to Figure 2, the latency time to fall in the CPZ and sham groups significantly decreased in comparison to the experimental groups. In the group treated with the high dose of extract, the latency increased significantly, and there was no significant difference between this group and the control group.

**Extract of ginger** **and fingolimod improved remyelination**

**Fig. 3:**
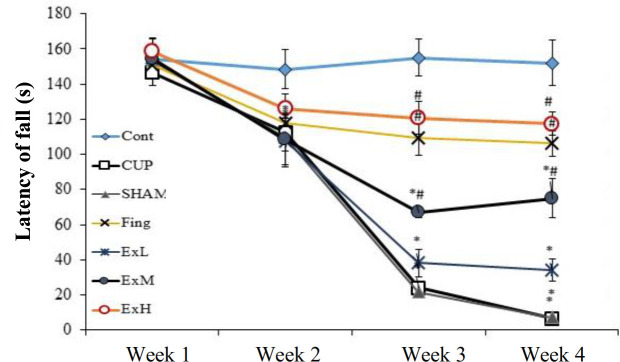
Transmission electron micrographs of CC of rats and the graph of myelin thickness of the studied groups. (A) Effect of ginger extract on remyelination of CC of the rats’ brains. The images was obtained by the TEM from the CC in the studied groups. Demyelination in the CPZ and sham groups, as well as the destruction of the demyelinated axons (DA) and vacuole formation (V) can be observed. In the ExH group, remyelination is performed properly (TEM 2000×). (B) The mean± SE of the thickness of myelin in the studied groups. Control group (Cont.), CUP group, sham group, Fingolimod group (Fing), fingolimod group; ExL, ExM, and ExH, *Zingiber officinale* extract groups (150, 300, and 600 mg/kg/day, respectively; The thickness of myelin in the fingolimod and ExH groups increased significantly compared to the CPZ and sham groups. ^*^Significant difference with the control group (*p *< 0.05); ^#^significant difference with the CPZ and sham groups (*p *< 0.05).

TEM revealed an increased thickness of myelinated axons in the CC in the high-dose extract group compared to the CPZ group (*p* < 0.05). The CUP group, sham group, and Fingolimod group showed no statistically significant difference, in relation to increase thickness of myelinated axons as observed in the control group. There was also no significant difference between the groups receiving high-dose extract and fingolimod (Fig. 3). Figure 3a displays the TEM of the CC of the rats’ brains in the studied groups. The demyelination of the CPZ group can be observed. In the high dose of the ginger extract group, remyelination was done properly (TEM 2000×). As Figure 3b shows, the thickness of myelin in the fingolimod and high-dose extract groups increased significantly compared with the CPZ and sham groups. 

**The expression of** **M** ***bp*** **and Olig2 genes** **increased in treatment groups**

**Fig. 4:**
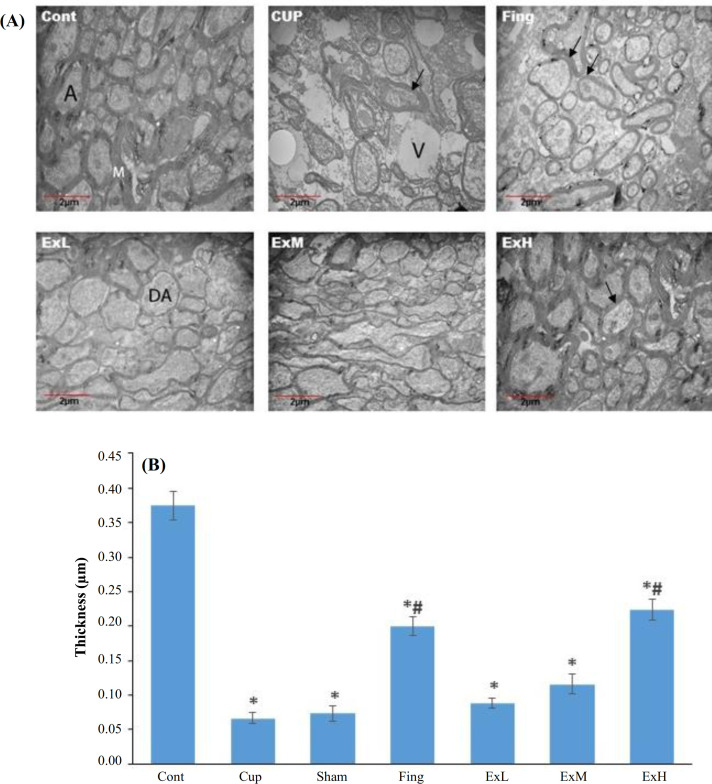
Effect of ginger extract on expression levels of Mbp (A) and Olig2 (B) genes. The figure shows mean ± SE of the gene expression levels in CC of studied groups. The expression levels of Mbp and Olig2 genes in the *Zingiber officinale* extract group (ExH) and fingolimod (Fing) groups significantly increased compared to other groups. Control group(Cont.), CUP group, sham group, fingolimod group (Fing); ExL, ExM, and ExH, *Zingiber officinale* extract groups (150, 300, and 600 mg/kg/day, respectively^ *^Significant difference with the control group; ^#^Significant difference with the CPZ and sham groups.

The results showed that the CPZ gavage caused a significant reduction (*p *< 0.001) in the M*bp* gene expression in rats with MS. The high dose of ginger extract and fingolimod consumption significantly (*p *< 0.001) increased the M*bp* expression level in treatment groups than in the CPZ groups, five weeks after administration. Medium and low doses of ginger increased the M*bp* expression level; nonetheless, no statistical difference was observed between the mentioned doses. In all experimental groups, the M*bp* gene expression increased in comparison to the CPZ and sham groups (Fig. 4A). There was no significant difference between the high-dose extract and fingolimod groups. The expression levels of Olig2 in all treatment groups were significantly (*p *< 0.05) higher than the CPZ group. In the current study, the Olig2 expression level increased in the fingolimod and the high dose of extract groups; however, it was statistically significant (*p* < 0.05) compared to the control group. Figure 4B shows the Olig2 gene expression level in the studied groups. In all experimental groups, the olig2 gene expression increased, except for the CPZ and sham groups. There was no significant difference between the high dose of ginger extract and the fingolimod groups. 

**MBP and Olig2** **protein levels increased in treatment groups**

**Fig. 5:**
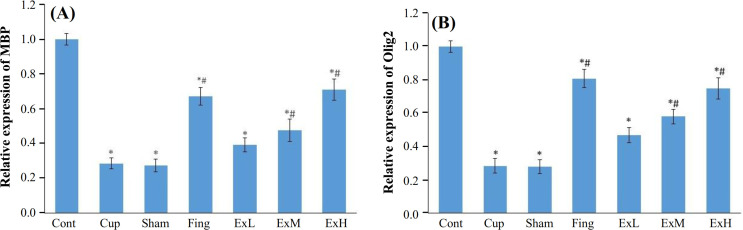
Effect of ginger extract on MBP (A) and Olig2 (B) protein levels. The OD of MBP and Olig2 protein in groups showed a significant difference between fingolimod and the high-dose of *Zingiber officinale* extract group and the CPZ and sham groups. Control group (Cont.), CUP group, sham group, Fingolimod group (Fing), ExL, ExM, and ExH, *Zingiber officinale* extract groups (150, 300, and 600 mg/kg/day, respectively; ^*^Significant difference with the control group; ^#^significant difference with the CPZ and sham groups. *: Significant difference with the control group. #: Significant difference with CPZ and sham groups.

The direct competitive ELISA technique was used to measure the protein level of MBP. In this method, OD has an inverse relationship with the antigen level of this protein. In the fingolimod group, the protein level of MBP increased and showed a difference from the control group. Also, the high-dose extract group indicated an increase in the protein level of MBP; however, it had no significant difference with the fingolimod group. Regarding the level of OD for MBP protein in groups, there was no significant difference between the fingolimod and high-dose extract groups (Fig. 5A). However, there was a significant difference between the treatment groups (fingolimod and a high dose of ginger extract) and CPZ groups. An indirect competitive ELISA technique was used to measure the protein level of Olig2. In this method, the OD has a direct relation with the amount of antigen. Accordingly, the level of Olig2 protein increased in all treated groups, except for the CPZ group. There were no significant differences between the three treatment groups of ginger extract. Regarding the level of OD for Olig2 protein in the groups, there was no significant difference between the fingolimod, high dose of ginger extract, and control groups (Figure 5B). However, there was a significant difference between the CPZ groups.

## DISCUSSION

MS is one of the most common neurological disorders in young people^[36]^. Inflammation and demyelination of the CNS are two main pathological events in MS^[37]^. Demyelinating diseases, such as MS, can be induced in the CNS of laboratory animals in different ways; for instance, toxin-induced demyelination^[38]^, EAE^[39,40]^, and virus-induced demyelination^[41,42]^. CPZ is a copper-chelating agent when administrated orally causes extensive demyelination in white or gray matter in the CNS. The demyelination induced by CPZ depends on unremitting exposure^[43,44]^. 

In the current study, CPZ was administered daily by oral gavage for four weeks to induce the MS model. Based on the results obtained by the electron microscopy and behavioral test, this model was well established in rats. Several studies have focused on different approaches to improve myelination, such as targeting specific signaling pathways, stem cell therapy, suppressing the inflammation process, and the reprogramming of glial cells to oligodendrocyte precursor cells^[45]^. Different drugs, such as glatiramer acetate, natalizumab, alemtuzumab, and fingolimod are currently used to cure MS^[46]^, which are relatively effective and their adverse effects make them unsuitable for prolonged use^[47]^. There is still no definitive cure for MS; therefore, trying to find an efficient and safe treatment seems to be essential^[48]^.

Over the last decade, alternative therapies for treating MS, especially herbal medicine, have noticeably been used in MS patients^[49,50]^. Today, several research trials have revealed that ginger and its bioactive components have antioxidant^[51,52]^ and neuro-protective^[32]^ effects. Ginger is used by MS patients due to its antiinflammatory activity^[53]^. In this study, the effect of different doses of extract of ginger was investigated on MS rat models and compared with the fingolimod group, in order to assess the effectiveness of ginger In a rat model of MS.

The present study found that the potential therapeutic effect of ginger extract at a dose of 600 mg/kg could prevent axonal demyelination in CC. There was no meaningful difference between the control and standard (fingolimod) groups, whereas a significant difference was observed between the CPZ-induced MS and experimental (600 mg/kg) groups. Medium and low doses of ginger extract also showed the same effects; however, these effects were not statistically significant. 

In the electron microscopic study, the high dose of ginger extract significantly protected the myelin sheath degeneration, which was confirmed by the behavioral test and reverse transcription-polymerase chain reaction and ELISA methods. Similar to our findings, Rittchen *et al.*^[54]^ indicated the useful and beneficial effects of ginger compounds in a vast range of inflammatory disorders, such as MS. Nanotherapy-based delivery of leukemia inhibitory factor directly to oligodendrocyte precursor cells proved to be highly potent in promoting myelin repair *in vivo*. This study revealed a novel approach of delivering drugs, which can lead to myelin repair in neuronal diseases^[54]^. Jafarzadeh *et al.*^[53]^ have reported the effectiveness of ginger on the immunological and inflammatory conditions in EAE. El-Akabawy and El-Kholy^[55]^ studied diabetes-induced CNS damage^[55]^ and exhibited that ginger had a potential therapeutic effect on this neuronal damage. **Mehdizadeh** *et al.*^[56]^ have demonstrated that ginger treatment can increase the number of neuronal cells experiencing MDMA-induced neurotoxicity. Also, the downregulation of Bcl-2 and upregulation of Bax were observed in ginger groups^[56]^. Gaire *et al.*^[57]^ have displayed that ginger compounds, such as 6-paradol, effectively protect the brain after cerebral ischemia, likely by attenuating neuroinflammation. 

In conclusion, our findings provide evidence that the administration of ginger extract prevents demyelination and improves remyelination in the CNS. Therefore, the ameliorative effect of *Zingiber officinale* extract, and its underlying mechanism of protective effect against some neurodegenerative diseases can play a role as a therapeutic agent in the management of MS.

## Acknowledgments

This study was extracted from a Ph.D. thesis by Valiollah Moradi in the Anatomical Science & Cell Biology Department. We are grateful to Prof. Masoud Etemadifar in Isfahan MS Research Center. We also thank the Deputy for Research of Isfahan University of Medical Sciences for supporting the research (Grant No.: 395262).

## Ethical statement

This research was approved by the Ethical Committee of Isfahan University of Medical Sciences (Ethics code: IR.MUI.REC.1395.3.262).

## Data availability

The data and materials of the study are available from the corresponding author on reasonable request.

## Author contributions

VM: contributed to conception and design of the experiment, performed all experiments, analyzed the data, and wrote, supervised and edited the manuscript; EE: contributed to conception and design of the experiment, helped complete all the experiments, and wrote, supervised and edited the manuscript; MG: helped to complete all the experiments; NG: helped to complete all the experiments; BR helped to complete all the experiments. All authors read and approved the final manuscript.

## Conflict of interest

None declared.

## Funding/support

The research was supported by Isfahan University of medical sciences, Isfahan, Iran.
